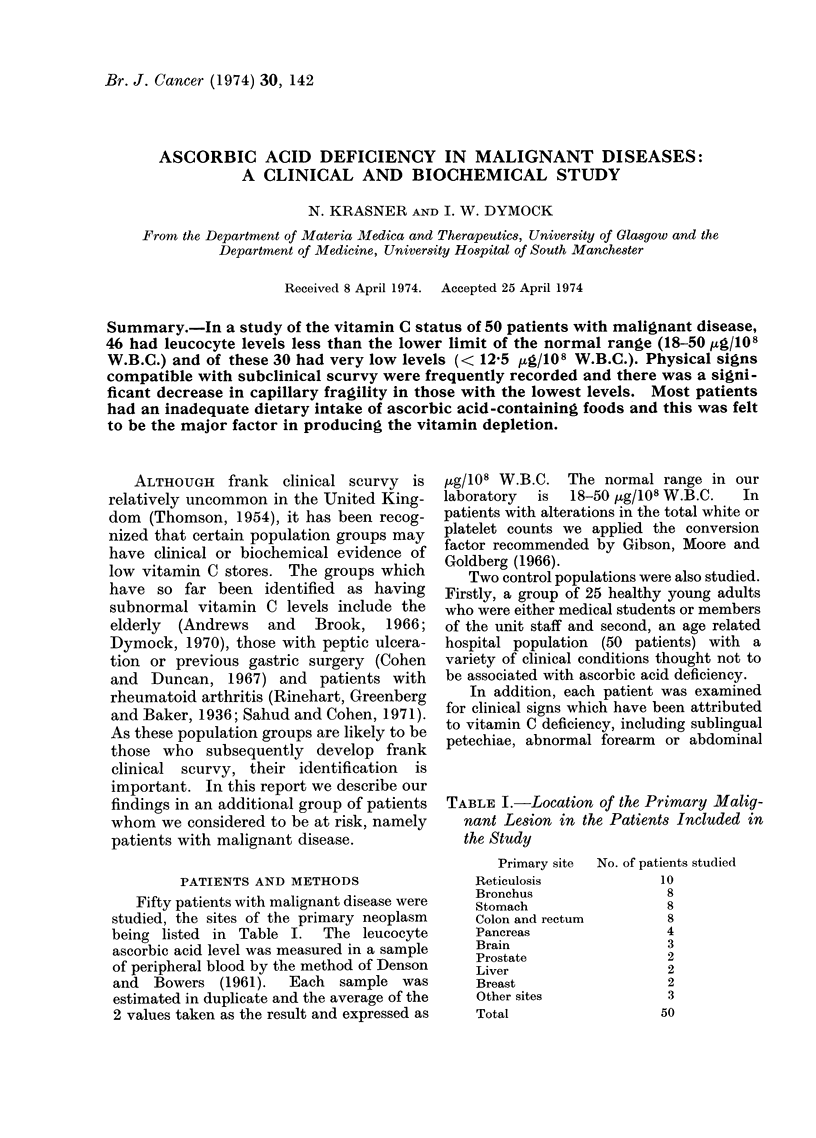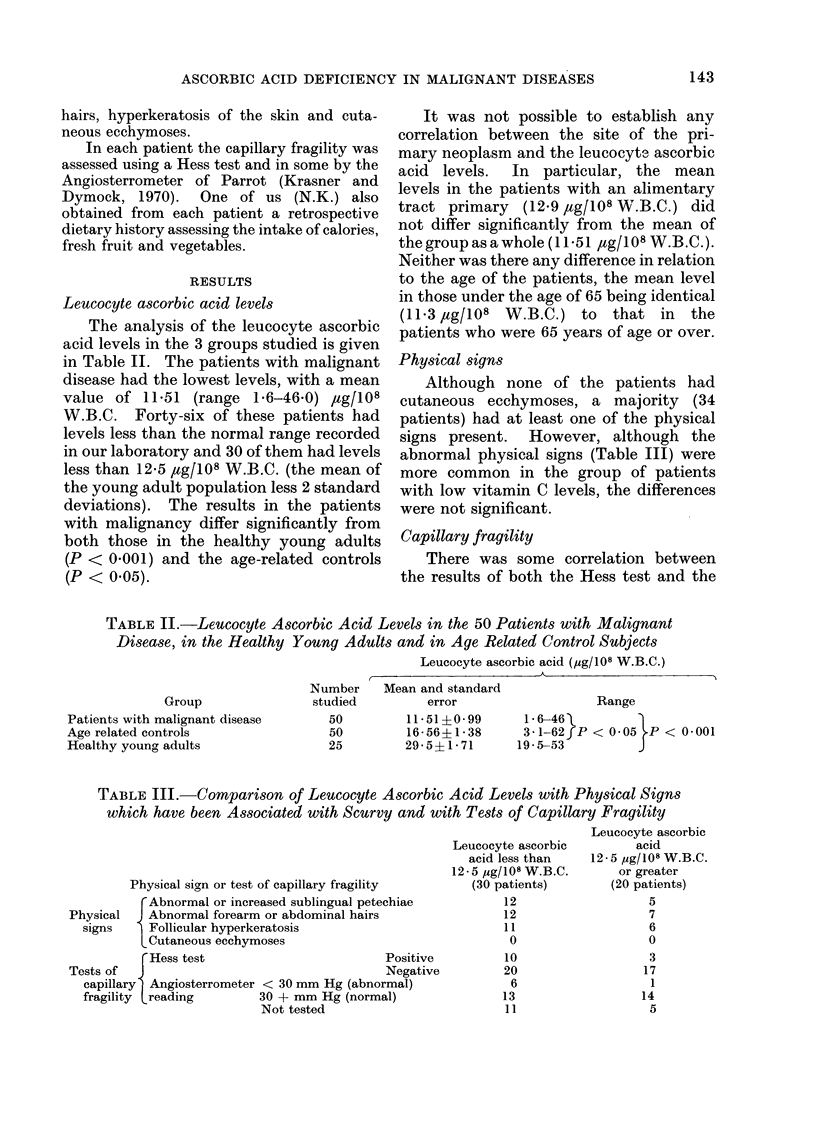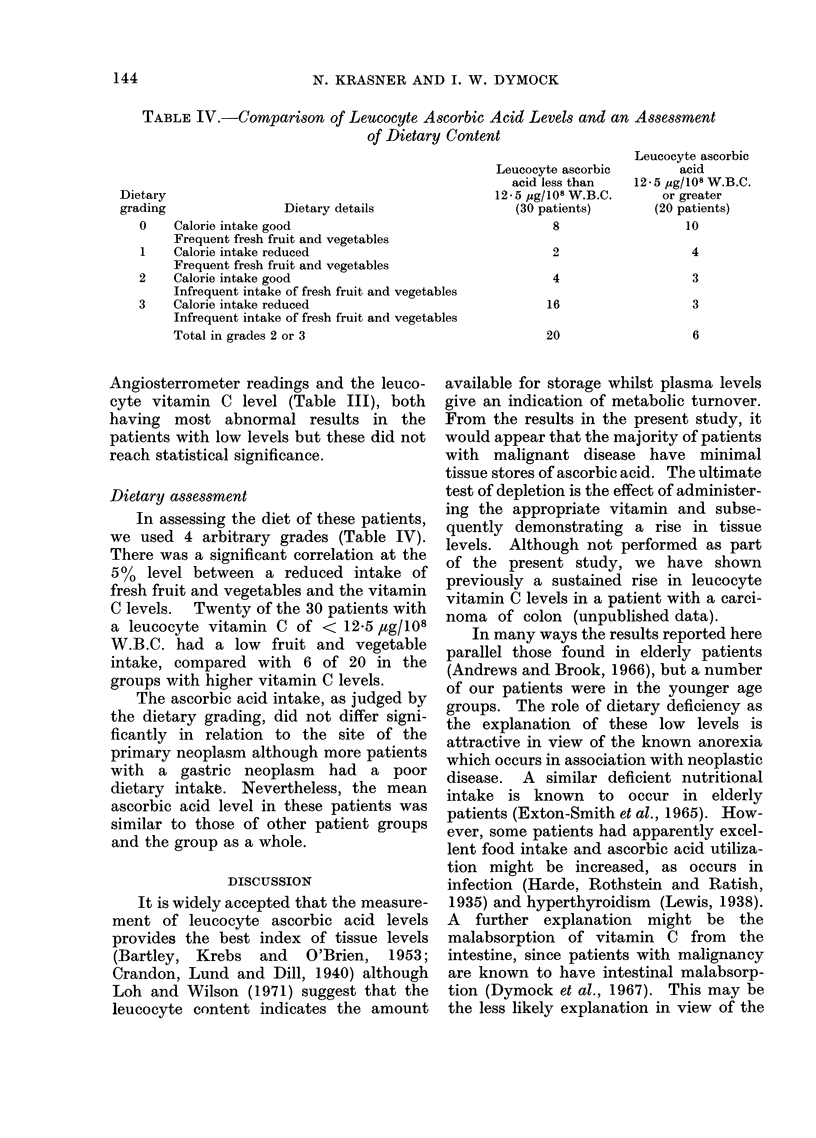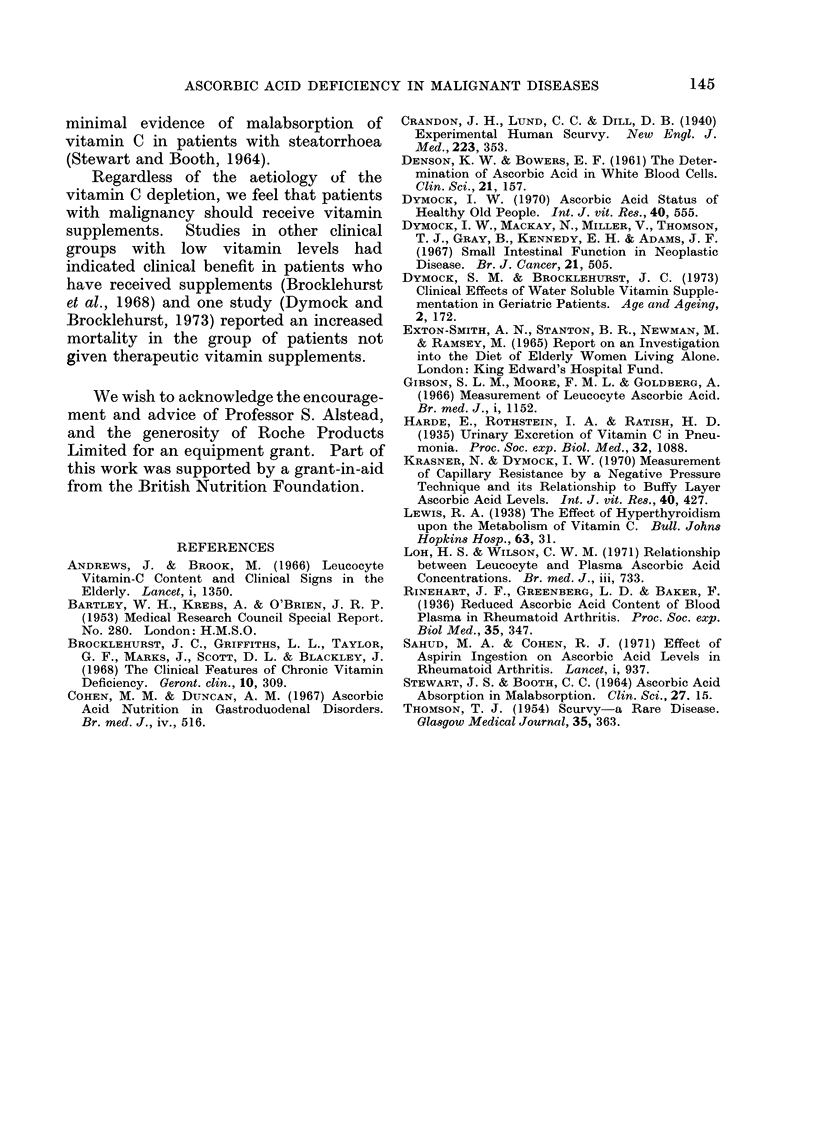# Ascorbic Acid Deficiency in Malignant Diseases: A Clinical and Biochemical Study

**DOI:** 10.1038/bjc.1974.125

**Published:** 1974-08

**Authors:** N. Krasner, I. W. Dymock

## Abstract

In a study of the vitamin C status of 50 patients with malignant disease, 46 had leucocyte levels less than the lower limit of the normal range (18-50,μg/10^8^ W.B.C.) and of these 30 had very low levels (< 12.5 μg/10^8^ W.B.C.). Physical signs compatible with subclinical scurvy were frequently recorded and there was a significant decrease in capillary fragility in those with the lowest levels. Most patients had an inadequate dietary intake of ascorbic acid-containing foods and this was felt to be the major factor in producing the vitamin depletion.


*Br. J. Cancer* (1974) **30**, 142

### ASCORBIC ACID DEFICIENCY IN MALIGNANT DISEASES: A CLINICAL AND BIOCHEMICAL STUDY

N. KRASNER AND I. W. DYMOCK

*From the Department of Materia Medica and Therapeutics, University of Glasgow and the Department of Medicine, University Hospital of South Manchester*

Received 8 April 1974. Accepted 25 April 1974

**Summary.—In a study of the vitamin C status of 50 patients with malignant disease, 46 had leucocyte levels less than the lower limit of the normal range (18–50 μg/$10^8$ W.B.C.) and of these 30 had very low levels (< 12·5 μg/$10^8$ W.B.C.). Physical signs compatible with subclinical scurvy were frequently recorded and there was a significant decrease in capillary fragility in those with the lowest levels. Most patients had an inadequate dietary intake of ascorbic acid-containing foods and this was felt to be the major factor in producing the vitamin depletion.**

ALTHOUGH frank clinical scurvy is relatively uncommon in the United Kingdom (Thomson, 1954), it has been recognized that certain population groups may have clinical or biochemical evidence of low vitamin C stores. The groups which have so far been identified as having subnormal vitamin C levels include the elderly (Andrews and Brook, 1966; Dymock, 1970), those with peptic ulceration or previous gastric surgery (Cohen and Duncan, 1967) and patients with rheumatoid arthritis (Rinehart, Greenberg and Baker, 1936; Sahud and Cohen, 1971). As these population groups are likely to be those who subsequently develop frank clinical scurvy, their identification is important. In this report we describe our findings in an additional group of patients whom we considered to be at risk, namely patients with malignant disease.

### PATIENTS AND METHODS

Fifty patients with malignant disease were studied, the sites of the primary neoplasm being listed in Table I. The leucocyte ascorbic acid level was measured in a sample of peripheral blood by the method of Denson and Bowers (1961). Each sample was estimated in duplicate and the average of the 2 values taken as the result and expressed as μg/$10^8$ W.B.C. The normal range in our laboratory is 18–50 μg/$10^8$ W.B.C. In patients with alterations in the total white or platelet counts we applied the conversion factor recommended by Gibson, Moore and Goldberg (1966).

Two control populations were also studied. Firstly, a group of 25 healthy young adults who were either medical students or members of the unit staff and second, an age related hospital population (50 patients) with a variety of clinical conditions thought not to be associated with ascorbic acid deficiency.

In addition, each patient was examined for clinical signs which have been attributed to vitamin C deficiency, including sublingual petechiae, abnormal forearm or abdominal

TABLE I.—*Location of the Primary Malignant Lesion in the Patients Included in the Study*

| Primary site | No. of patients studied |
|---|---|
| Reticulosis | 10 |
| Bronchus | 8 |
| Stomach | 8 |
| Colon and rectum | 8 |
| Pancreas | 4 |
| Brain | 3 |
| Prostate | 2 |
| Liver | 2 |
| Breast | 2 |
| Other sites | 3 |
| Total | 50 |

hairs, hyperkeratosis of the skin and cutaneous ecchymoses.

In each patient the capillary fragility was assessed using a Hess test and in some by the Angiosterrometer of Parrot (Krasner and Dymock, 1970). One of us (N.K.) also obtained from each patient a retrospective dietary history assessing the intake of calories, fresh fruit and vegetables.

### RESULTS

### *Leucocyte ascorbic acid levels*

The analysis of the leucocyte ascorbic acid levels in the 3 groups studied is given in Table II. The patients with malignant disease had the lowest levels, with a mean value of 11·51 (range 1·6–46·0) μg/$10^8$ W.B.C. Forty-six of these patients had levels less than the normal range recorded in our laboratory and 30 of them had levels less than 12·5 μg/$10^8$ W.B.C. (the mean of the young adult population less 2 standard deviations). The results in the patients with malignancy differ significantly from both those in the healthy young adults ($P < 0{\cdot}001$) and the age-related controls ($P < 0{\cdot}05$).

It was not possible to establish any correlation between the site of the primary neoplasm and the leucocyte ascorbic acid levels. In particular, the mean levels in the patients with an alimentary tract primary (12·9 μg/$10^8$ W.B.C.) did not differ significantly from the mean of the group as a whole (11·51 μg/$10^8$ W.B.C.). Neither was there any difference in relation to the age of the patients, the mean level in those under the age of 65 being identical (11·3 μg/$10^8$ W.B.C.) to that in the patients who were 65 years of age or over.

### *Physical signs*

Although none of the patients had cutaneous ecchymoses, a majority (34 patients) had at least one of the physical signs present. However, although the abnormal physical signs (Table III) were more common in the group of patients with low vitamin C levels, the differences were not significant.

### *Capillary fragility*

There was some correlation between the results of both the Hess test and the

TABLE II.—*Leucocyte Ascorbic Acid Levels in the 50 Patients with Malignant Disease, in the Healthy Young Adults and in Age Related Control Subjects*

| Group | Number studied | Leucocyte ascorbic acid (μg/$10^8$ W.B.C.): Mean and standard error | Range | | |
|---|---|---|---|---|---|
| Patients with malignant disease | 50 | 11·51±0·99 | 1·6–46 | $P < 0{\cdot}05$ (first two groups) | $P < 0{\cdot}001$ (all three groups) |
| Age related controls | 50 | 16·56±1·38 | 3·1–62 | | |
| Healthy young adults | 25 | 29·5±1·71 | 19·5–53 | | |

TABLE III.—*Comparison of Leucocyte Ascorbic Acid Levels with Physical Signs which have been Associated with Scurvy and with Tests of Capillary Fragility*

| | Physical sign or test of capillary fragility | | Leucocyte ascorbic acid less than 12·5 μg/$10^8$ W.B.C. (30 patients) | Leucocyte ascorbic acid 12·5 μg/$10^8$ W.B.C. or greater (20 patients) |
|---|---|---|---|---|
| Physical signs | Abnormal or increased sublingual petechiae | | 12 | 5 |
| | Abnormal forearm or abdominal hairs | | 12 | 7 |
| | Follicular hyperkeratosis | | 11 | 6 |
| | Cutaneous ecchymoses | | 0 | 0 |
| Tests of capillary fragility | Hess test | Positive | 10 | 3 |
| | | Negative | 20 | 17 |
| | Angiosterrometer reading | < 30 mm Hg (abnormal) | 6 | 1 |
| | | 30 + mm Hg (normal) | 13 | 14 |
| | | Not tested | 11 | 5 |

TABLE IV.—*Comparison of Leucocyte Ascorbic Acid Levels and an Assessment of Dietary Content*

| Dietary grading | Dietary details | Leucocyte ascorbic acid less than 12·5 $\mu g/10^8$ W.B.C. (30 patients) | Leucocyte ascorbic acid 12·5 $\mu g/10^8$ W.B.C. or greater (20 patients) |
|---|---|---|---|
| 0 | Calorie intake good<br>Frequent fresh fruit and vegetables | 8 | 10 |
| 1 | Calorie intake reduced<br>Frequent fresh fruit and vegetables | 2 | 4 |
| 2 | Calorie intake good<br>Infrequent intake of fresh fruit and vegetables | 4 | 3 |
| 3 | Calorie intake reduced<br>Infrequent intake of fresh fruit and vegetables | 16 | 3 |
| | Total in grades 2 or 3 | 20 | 6 |

Angiosterrometer readings and the leucocyte vitamin C level (Table III), both having most abnormal results in the patients with low levels but these did not reach statistical significance.

### *Dietary assessment*

In assessing the diet of these patients, we used 4 arbitrary grades (Table IV). There was a significant correlation at the 5% level between a reduced intake of fresh fruit and vegetables and the vitamin C levels. Twenty of the 30 patients with a leucocyte vitamin C of $< 12{\cdot}5\ \mu g/10^8$ W.B.C. had a low fruit and vegetable intake, compared with 6 of 20 in the groups with higher vitamin C levels.

The ascorbic acid intake, as judged by the dietary grading, did not differ significantly in relation to the site of the primary neoplasm although more patients with a gastric neoplasm had a poor dietary intake. Nevertheless, the mean ascorbic acid level in these patients was similar to those of other patient groups and the group as a whole.

### DISCUSSION

It is widely accepted that the measurement of leucocyte ascorbic acid levels provides the best index of tissue levels (Bartley, Krebs and O'Brien, 1953; Crandon, Lund and Dill, 1940) although Loh and Wilson (1971) suggest that the leucocyte content indicates the amount available for storage whilst plasma levels give an indication of metabolic turnover. From the results in the present study, it would appear that the majority of patients with malignant disease have minimal tissue stores of ascorbic acid. The ultimate test of depletion is the effect of administering the appropriate vitamin and subsequently demonstrating a rise in tissue levels. Although not performed as part of the present study, we have shown previously a sustained rise in leucocyte vitamin C levels in a patient with a carcinoma of colon (unpublished data).

In many ways the results reported here parallel those found in elderly patients (Andrews and Brook, 1966), but a number of our patients were in the younger age groups. The role of dietary deficiency as the explanation of these low levels is attractive in view of the known anorexia which occurs in association with neoplastic disease. A similar deficient nutritional intake is known to occur in elderly patients (Exton-Smith *et al.*, 1965). However, some patients had apparently excellent food intake and ascorbic acid utilization might be increased, as occurs in infection (Harde, Rothstein and Ratish, 1935) and hyperthyroidism (Lewis, 1938). A further explanation might be the malabsorption of vitamin C from the intestine, since patients with malignancy are known to have intestinal malabsorption (Dymock *et al.*, 1967). This may be the less likely explanation in view of the minimal evidence of malabsorption of vitamin C in patients with steatorrhoea (Stewart and Booth, 1964).

Regardless of the aetiology of the vitamin C depletion, we feel that patients with malignancy should receive vitamin supplements. Studies in other clinical groups with low vitamin levels had indicated clinical benefit in patients who have received supplements (Brocklehurst *et al.*, 1968) and one study (Dymock and Brocklehurst, 1973) reported an increased mortality in the group of patients not given therapeutic vitamin supplements.

We wish to acknowledge the encouragement and advice of Professor S. Alstead, and the generosity of Roche Products Limited for an equipment grant. Part of this work was supported by a grant-in-aid from the British Nutrition Foundation.

### REFERENCES

ANDREWS, J. & BROOK, M. (1966) Leucocyte Vitamin-C Content and Clinical Signs in the Elderly. *Lancet*, i, 1350.

BARTLEY, W. H., KREBS, A. & O'BRIEN, J. R. P. (1953) Medical Research Council Special Report. No. 280. London: H.M.S.O.

BROCKLEHURST, J. C., GRIFFITHS, L. L., TAYLOR, G. F., MARKS, J., SCOTT, D. L. & BLACKLEY, J. (1968) The Clinical Features of Chronic Vitamin Deficiency. *Geront. clin.*, **10**, 309.

COHEN, M. M. & DUNCAN, A. M. (1967) Ascorbic Acid Nutrition in Gastroduodenal Disorders. *Br. med. J.*, iv., 516.

CRANDON, J. H., LUND, C. C. & DILL, D. B. (1940) Experimental Human Scurvy. *New Engl. J. Med.*, **223**, 353.

DENSON, K. W. & BOWERS, E. F. (1961) The Determination of Ascorbic Acid in White Blood Cells. *Clin. Sci.*, **21**, 157.

DYMOCK, I. W. (1970) Ascorbic Acid Status of Healthy Old People. *Int. J. vit. Res.*, **40**, 555.

DYMOCK, I. W., MACKAY, N., MILLER, V., THOMSON, T. J., GRAY, B., KENNEDY, E. H. & ADAMS, J. F. (1967) Small Intestinal Function in Neoplastic Disease. *Br. J. Cancer*, **21**, 505.

DYMOCK, S. M. & BROCKLEHURST, J. C. (1973) Clinical Effects of Water Soluble Vitamin Supplementation in Geriatric Patients. *Age and Ageing*, **2**, 172.

EXTON-SMITH, A. N., STANTON, B. R., NEWMAN, M. & RAMSEY, M. (1965) Report on an Investigation into the Diet of Elderly Women Living Alone. London: King Edward's Hospital Fund.

GIBSON, S. L. M., MOORE, F. M. L. & GOLDBERG, A. (1966) Measurement of Leucocyte Ascorbic Acid. *Br. med. J.*, i, 1152.

HARDE, E., ROTHSTEIN, I. A. & RATISH, H. D. (1935) Urinary Excretion of Vitamin C in Pneumonia. *Proc. Soc. exp. Biol. Med.*, **32**, 1088.

KRASNER, N. & DYMOCK, I. W. (1970) Measurement of Capillary Resistance by a Negative Pressure Technique and its Relationship to Buffy Layer Ascorbic Acid Levels. *Int. J. vit. Res.*, **40**, 427.

LEWIS, R. A. (1938) The Effect of Hyperthyroidism upon the Metabolism of Vitamin C. *Bull. Johns Hopkins Hosp.*, **63**, 31.

LOH, H. S. & WILSON, C. W. M. (1971) Relationship between Leucocyte and Plasma Ascorbic Acid Concentrations. *Br. med. J.*, iii, 733.

RINEHART, J. F., GREENBERG, L. D. & BAKER, F. (1936) Reduced Ascorbic Acid Content of Blood Plasma in Rheumatoid Arthritis. *Proc. Soc. exp. Biol Med.*, **35**, 347.

SAHUD, M. A. & COHEN, R. J. (1971) Effect of Aspirin Ingestion on Ascorbic Acid Levels in Rheumatoid Arthritis. *Lancet*, i, 937.

STEWART, J. S. & BOOTH, C. C. (1964) Ascorbic Acid Absorption in Malabsorption. *Clin. Sci.*, **27**, 15.

THOMSON, T. J. (1954) Scurvy—a Rare Disease. *Glasgow Medical Journal*, **35**, 363.